# A Microbial-Based Approach to Mental Health: The Potential of Probiotics in the Treatment of Depression

**DOI:** 10.3390/nu15061382

**Published:** 2023-03-13

**Authors:** Dinyadarshini Johnson, Vengadesh Letchumanan, Chern Choong Thum, Sivakumar Thurairajasingam, Learn-Han Lee

**Affiliations:** 1 Novel Bacteria and Drug Discovery Research Group (NBDD), Microbiome and Bioresource Research Strength (MBRS), Jeffrey Cheah School of Medicine and Health Sciences, Monash University Malaysia, Bandar Sunway 47500, Malaysia; 2 Pathogen Resistome Virulome and Diagnostic Research Group (PathRiD), Jeffrey Cheah School of Medicine and Health Sciences, Monash University Malaysia, Bandar Sunway 47500, Malaysia; 3 Department of Psychiatry, Hospital Sultan Abdul Aziz Shah, Persiaran Mardi-UPM, Serdang 43400, Malaysia; 4 Clinical School Johor Bahru, Jeffrey Cheah School of Medicine and Health Sciences, Monash University Malaysia, Johor Bahru 80100, Malaysia

**Keywords:** probiotics, depression, psychiatry, precision, gut microbes, major depressive disorder

## Abstract

Probiotics are currently the subject of intensive research pursuits and also represent a multi-billion-dollar global industry given their vast potential to improve human health. In addition, mental health represents a key domain of healthcare, which currently has limited, adverse-effect prone treatment options, and probiotics may hold the potential to be a novel, customizable treatment for depression. Clinical depression is a common, potentially debilitating condition that may be amenable to a precision psychiatry-based approach utilizing probiotics. Although our understanding has not yet reached a sufficient level, this could be a therapeutic approach that can be tailored for specific individuals with their own unique set of characteristics and health issues. Scientifically, the use of probiotics as a treatment for depression has a valid basis rooted in the microbiota-gut-brain axis (MGBA) mechanisms, which play a role in the pathophysiology of depression. In theory, probiotics appear to be ideal as adjunct therapeutics for major depressive disorder (MDD) and as stand-alone therapeutics for mild MDD and may potentially revolutionize the treatment of depressive disorders. Although there is a wide range of probiotics and an almost limitless range of therapeutic combinations, this review aims to narrow the focus to the most widely commercialized and studied strains, namely *Lactobacillus* and *Bifidobacterium*, and to bring together the arguments for their usage in patients with major depressive disorder (MDD). Clinicians, scientists, and industrialists are critical stakeholders in exploring this groundbreaking concept.

## 1. Introduction

Probiotics were first defined by Elie Metchnikoff, a Nobel laureate in the early 1900s, and over time leading up to the present, the field of probiotic research has expanded tremendously, with a range of work supporting their vast and far-reaching health benefits. The perception of probiotics has also changed from being viewed as an overhyped remedy a century ago to its acceptance as an over-the-counter supplement, dietary product, and therapeutic drug today. In terms of therapeutics, the current research efforts are moving towards identifying strain-specific and disease-specific probiotics to optimize probiotics therapeutic potential [1,2,3,4,5,6]. The promising outcomes of probiotic intervention have been, by and large, ascertained in pre-clinical models. Probiotics’ potentially therapeutic roles have been explored in various medical domains, including psychiatric, gastrointestinal, cardio-metabolic, dermatological, neurological, gynecological, and oncological domains [2,4,5]. The potential of probiotics to produce desired health outcomes amid the highly heterogeneous human gut microbiome and possible epigenetic interference is probably the most intriguing aspect of any probiotic intervention in the human population [7].

Probiotic use as a potential therapeutic modality in psychiatry has been gradually advancing since the early 2000s [8]. Understanding the bidirectional interaction between the human brain and the gut microbiota within the microbiota-gut-brain axis (MGBA) has paved the way to apprehend the potentially beneficial role of probiotics in the elusive and relatively poorly understood field of mental health [1,2,3,9]. Nevertheless, some critical historical precursors to this scientific niche can be traced back to the 19th century. The foremost hypothetical correlation between the gut and systemic health was postulated by a French physician, Charles Bouchard (1837–1915), through his theory of autointoxication. This theory was founded on the belief that the retention of intestinal waste is poisonous to the body and ascribed countless ailments, most notably in the mental realm, to the ‘curse’ of constipation, thus vaguely establishing the connection between the gut and the mental faculty [9,10]. Emmanuel Régis, Antónoio Mario de Bettencourt Rodrigues, and François-André Chevalier-Lavaure were among the conceptual precursors who acknowledged the possible role of intestinal microbiota in mental health concerning autointoxication. Rodrigues’s publication linking depression and melancholia to gastrointestinal autointoxication blazed a trail for exploring intestinal microbiota with regard to the mental realm [9,11,12]. These theoretical rudiments of intestinal microbiota remained scientifically unfounded until 1907, when the ingenious and avid experimenter Metchnikoff emerged as the pioneering advocate of probiotics. He attributed the rural Bulgarians’ enhanced longevity and delayed senility to their regular consumption of lactic acid bacteria in fermented dairy products. Metchnikoff’s discovery spurred the widespread commercialization of *Lactobacillus* formulations as the panacea for physical and mental ailments. His landmark notion of ‘fighting microbes with microbes’ in 1912 aggrandized *L. bulgaricus* as the sovereign clinical remedy, replacing the radical treatment of colectomies and enemas. Subsequently, *L. acidophilus*, an indigenous gut microbe, emerged as a better alternative to Metchnikoff’s *Bulgaricus* and dominated the market as a treatment for autointoxication sequelae during the early decades of the 20th century. Melancholy, malaise, insomnia, diminished interest, and neuroses were some characteristics of depressive disorder deemed curable with ingesting *Lactobacillus* formulations [1,13].

Depression is among the neuropsychiatric disorders that have been extensively studied in relation to probiotics. It is one of the most common psychiatric disorders, affecting approximately 322 million people worldwide [13]. Recognizing depression as a global health concern, especially with the recent spike in its overall prevalence following the COVID-19 pandemic spell, probiotics offer a ray of hope to tackle this debilitating and potentially fatal disorder [2]. The first publication evaluating the possible beneficial role of probiotics in depression appeared in 2005 [8]. Probiotics have been demonstrated to possess comparable effectiveness to antidepressants. Additionally, probiotics have favorable side-effect profiles and no associated stigma barriers [2,14,15,16]. The beneficial effects of probiotics on depression have been well substantiated in recent systematic reviews and meta-analyses [17,18,19,20,21,22,23]. Most of the existing reviews have postulated the anti-depressive effects of probiotics based on the intertwined MGBA mechanisms in the pathophysiological occurrence of depression [2,20,24,25,26,27]. *Lactobacillus* spp. and *Bifidobacterium* spp. are the most widely studied probiotics in depression [26,28]. The dynamicity and efficacy of probiotics evoke the question of whether probiotics could be an ideal medicament that befits the emerging medical model of precision psychiatry [29,30,31].

Precision psychiatry is a branch of precision medicine. President Obama launched the “Precision Medicine Initiative” in 2015 to foster an integrated clinical approach that considers individual variability in disease prevention, diagnosis, and treatment strategies by utilizing advances in science and technology to tailor personalized medical care [29,32]. Since then, the field of medicine has seen a plethora of powerful advancements in diagnostic and therapeutic methods, from the development of large-volume biologic databases to the incorporation of machine learning that leverages multi-modal algorithms in disease and outcome prediction [33,34]. Precision medicine, however, is not entirely an alien concept and has always been an integral part of clinical practice. Archibald Garrod is regarded as the father of precision medicine. His remarkable work, published in 1902, highlighted the chemical variations in individuals and the clinicians’ role in providing personalized care [35]. Personalized care is the ultimate goal of precision medicine. However, there are limitations in consolidating the existing ocean of knowledge owing to the discrepancies between various clinical specialties and significant translational gaps [32,35]. Unlike other branches of medicine, the term “precision” has a different meaning in the psychiatric domain, considering the complexity and challenges associated with the clinical approach to psychiatric disorders. Diagnosing psychiatric disorders largely depends on clinical expertise and lacks diagnostic laboratory and imaging assessments. Hence, the core application of the precision psychiatry model is often advocated by considering the possible underlying neurobiological mechanisms and environmental and lifestyle influences in the development of a psychiatric disorder for treatment optimization [32,35,36,37,38,39]. The microbial-based approach unfolds a new horizon in the therapeutic landscape and management of psychiatric disorders by accommodating the precision psychiatry concept through the personalization of probiotics, which has been recommended in several studies [6,40]. The integration of the conceptual components of precision psychiatry-neurobiological underpinnings, genetic, environmental, and lifestyle variabilities—and the exploration of potential microbial-based assessments to demonstrate the potential of probiotics in depression—is a consolidated attempt to embrace this revolutionary model of psychiatric care and to mitigate the existing translational gap. Although we are only at the tip of the iceberg, the work that has been done towards evaluating probiotics’ clinical applicability in alignment with the contemporary healthcare model echoes the timely need to recognize this promising therapeutic targeting a global concern, depression [35,41,42,43,44,45]. Therefore, in this review, we are attempting an evolutionary approach to deduce a conceptual framework of probiotics befitting the precision psychiatry model of healthcare by paying particular heed to the anti-depressive potential of the most widely studied and commercialized *Lactobacillus* and *Bifidobacterium* probiotics, coupled with relevant examinations of the MGBA.

## 2. Rudiments of Probiotics: Human Gut Microbiota and Gut Dysbiosis

Probiotics are essentially microbes, commonly bacteria, with either human or non-human origins [46,47,48]. Although probiotics have been in use for ages, the term ‘probiotics’ appeared only in 1953, courtesy of German scientist Werner Kollath. A consensus defining probiotics as ‘live microorganisms that, when administered in adequate amounts, confer a health benefit on the host’ was established in 2013 by an expert panel convened by the International Scientific Association for Probiotics and Prebiotics (ISAPP) [1,2,9,49,50]. To explore the potential of probiotics in human health, it is essential to have a background understanding of the human microbiota. The term human microbiota collectively refers to all the microorganisms living in and on the human body as symbionts, including bacteria, archaea, viruses, fungi, and protists. However, this term is conventionally used to refer to bacteria due to its large order of magnitude compared to other microbiota in the human body [51]. The widely quoted fact that the microbiota outnumbers human cells at a ratio of 10:1 has been recently revised and updated to an estimated ratio of 1.3:1 with 3.8 × 10^13^ bacteria and 3.0 × 10^13^ human cells [52,53]. The colon is the largest reservoir of bacteria; hence, it is taken as the sole numerical contributor to the overall microbial population estimates in the human body. Although microbiota exist in a ratio equivalent to that of human cells, they make up only 0.3% of the total body mass due to their tiny volume [52,54,55]. Nevertheless, the role of microbiota in human physiology and pathophysiology is immense and indisputable, to the extent that a portion of the scientific community has acknowledged the gut microbiota as an organ in its own right [56,57,58,59,60,61].

Microbiota are first introduced in the human gut at birth through vertical transmission, although there are debatable claims that this could occur *in utero.* Mode of delivery, diet, and antibiotic exposure are crucial determinants of the pioneer colonizers of the infant’s gut [62]. The human gut is home to six main phyla of microbiota: *Firmicutes*, *Bacteroidetes*, *Actinobacteria*, *Proteobacteria*, *Fusobacteria*, and *Verrucomicrobia*, with *Firmicutes* and *Bacteroidetes* accounting for more than 90% of the gut microbiota. There are approximately 1000 bacterial species and over 7000 strains in the gut. *Lactobacillus* and *Bifidobacterium* are among the human gut’s dominant natural residents, particularly in the early years of life; they belong to the phyla Firmicutes and Actinobacteria, respectively. Beneficial *Lactobacillus* spp. and *Bifidobacterium* spp. are abundant in the early fecal samples of vaginal birth infants, with the latter strongly associated with breastfed infants [5,63,64,65,66,67,68]. At the same time, early colonization is critical in shaping an individual’s gut microbiota composition and health outcome. However, the sustainability of the baseline microbial pattern becomes a matter of concern owing to the possibly harmful interference of various internal and external stimuli at any given stage of life [64,68,69]. An untoward perturbation of the baseline gut microbial composition favors the pathogenic activity of the indigenous symbionts of the gut, hence giving rise to pathobionts that may, in turn, adversely affect the host’s physiology [70,71,72,73,74,75].

Microbiota possess a dynamic yet complex relationship with the human host, directly or indirectly via their by-products. These symbionts of the human host may interact as commensals, opportunistic pathobionts, or beneficial probiotics. Bidirectional communication between the gut and the brain is mediated by the gut microbiota, which involves the central nervous system, autonomic nervous system, and enteric nervous system within the MGBA. This gut microbiota-mediated pathway influences neurobehavioral outcomes through neuronal, endocrinal, and immunological mechanisms [76,77,78]. Microbiota co-evolve across an individual’s life span through a dynamic interplay with internal and external determinants. This interplay has also been regarded as an epigenetic mechanism incorporating a large body of internal and external factors that can exert a reversible change in the genetic expression without altering the actual deoxyribonucleic acid (DNA) sequence. The gut microbiota is the primary embodiment of communication within the gut-brain loop. [2,18,79,80]. Gut dysbiosis is a general term to refer to the significant adverse alteration in an individual’s gut microbial composition, negatively affecting the host’s health [78]. Ultimately, restoring gut dysbiosis specific to the associated health condition is the principal therapeutic goal of microbial-based applications.

In the context of depression, gut dysbiosis in human subjects with depression has been scientifically verified. The possibility of microbial dysbiosis as a possible cause of depressive disorder, however, remains a puzzle not yet fully understood. Nevertheless, more recent findings have substantiated the causative role of gut microbes in depression [81,82]. One of the current large-scale, population-based cohort studies explored the genome-wide association of the gut microbiome with depression in 2593 Europeans who were free of antidepressant use at the time of study in an attempt to establish the causal role of the gut microbiome in depression. Twelve genera: *Subdoligranulum*, *Coprococcus*, *Sellimonas*, *Ruminococcaceae* (*UCG002*, *UCG003*, and *UCG005*), *LachnospiraceaeUCG001*, *Eubacterium ventriosum*, and one family: *Ruminococcaceae* were negatively associated with depressive symptoms, while *Lachnoclostridium*, *Hungatella*, *Eggerthella*, and *Ruminococcusgauvreauiigroup* were positively related to depressive symptoms. Alpha diversity was negatively associated with depression. Following the Mendelian randomization analysis, *Eggerthella* was causally associated with MDD. All 13 taxa significantly associated with depression are involved in synthesizing various neurotransmitters, which are vital in depressive disorders [83]. Generally, the gist of gut dysbiosis findings in depressed human subjects as opposed to their healthy counterparts includes a lack of microbial diversity and an inversely proportional abundance of pathobionts to beneficial microbiota in the former [2,68,77,84]. However, there are observable discrepancies and inconsistencies among the reported findings regarding identified bacterial genera and species, alpha and beta diversities, and the overall pattern of gut dysbiosis [85]. One commonly reported result includes a significant disturbance in the equilibria of *Firmicutes* and *Bacteroidetes*, mostly a reduced abundance of *Firmicutes* and an increased abundance of *Bacteroidetes*, *Actinobacteria*, and *Proteobacteria* [23,77,84,86,87]. Only a handful of studies exclusively explored the proportions of the phylum *Firmicutes* and genera *Lactobacillus* and *Bifidobacterium* in depressed subjects, providing further attestation to the reduced abundance of these hallmark members of the human gut with depression [88,89]. *Lactobacillus* and *Bifidobacterium* counts were significantly lower in patients with MDD compared to healthy counterparts [89]. Although it appears to be a Herculean task to derive a standardized pattern of gut microbial dysbiosis, the existing findings of gut dysbiosis in depressed subjects have undoubtedly laid the foundation for understanding mechanisms implicating gut microbiota in the etiopathology of depression. Disruptions in the physiological renderings of the gut microbiota in maintaining the integrity of the gut membrane, modulation of inflammatory precursors and neurotransmitters, and regulation of the hypothalamic-pituitary-adrenal axis, either directly or indirectly, have been implicated in the pathophysiological occurrence of depression [2,77,90].

The role of probiotics in treating depression is essentially to restore the gut microbial balance by modulating the gut microbiota [2,4,7,91]. Ingestion of probiotics positively impacts human health by promoting the biotherapeutic activity of the beneficial microbiota while suppressing the pathogenic action of the pathobionts [70,92]. The ability to reach the target organ, commonly the intestines, survive various physiological stressors, including the varying pH down the gastrointestinal tract, and interrupt the disease’s pathogenesis make a microbe an ideal probiotic candidate [93,94]. Due to their diverse health-promoting properties, *Lactobacillus* spp. and *Bifidobacterium* spp. are the most common microbes used as probiotics. These bacterial species are known for their anti-depressive potential and favorable safety profiles. They are associated with low pathogenicity and barely potentiate horizontal transmission of antibiotic resistance to pathogens [26,78,95,96,97,98]. *Lactobacillus* and *Bifidobacterium* genera are composed of gram-positive, anaerobic bacterial species and are among the pioneer colonizers of the human gut. These catalase-negative bacteria produce lactic acid as their primary metabolic end-product of carbohydrate fermentation and confer abilities to survive the various physiological stressors down the gastrointestinal tract, key features qualifying them as preferred probiotic candidates [27,99,100,101,102]. The probiotic nomenclature begins with identifying its genus, species, subspecies (if applicable), and strain, which comes with an alphanumeric designation of the probiotic species, i.e., *Lactobacillus casei* DN-114 001 [103]. The identification of probiotics based on disease-specific and strain-specific efficacy caters to the application of probiotics in the precision psychiatry healthcare model [43,44,104,105]. The fostering of depression-specific probiotics requires examining the neurobiological, genetic, environmental, and lifestyle components involved in the development of depression that could be conceivably ameliorated through the use of probiotics. Exploring microbial-based clinical markers and integrated translation using machine learning specific to clinical depression strengthens the notion of probiotics as a valuable element of precision psychiatry [32,42,83,104,106].

## 3. A Conceptual Framework of Probiotics Aligned to Precision Psychiatry in Clinical Depression

Precision psychiatry has gained momentum in recent years with the development of more robust and validated prediction models tailored individually for diagnostic, prognostic, and treatment-response estimations. However, expanding knowledge and research initiatives in this domain are primarily limited by the need for practical implementation in real-world psychiatric practice [32,107]. One of the ways to bridge the translational gap is by developing consolidated frameworks for implementation purposes that are instrumental in embracing this revolutionary model of psychiatric care. The conceptual components of precision psychiatry refer to the neurobiological, genetic, environmental, and lifestyle bases simultaneously explored in relation to depression, which then link to the potential beneficial roles of probiotics. These components are derivatives of the precision medicine approach, which has conveniently been an integral part of other clinical specialties with lesser translational gaps compared to psychiatric specialties [35,107,108]. To further promote the integration of this concept within the clinical context, the potential and futuristic microbial-based clinical approaches have also been explored and identified in support of prediction modeling. Understanding the existing clinical approach toward the target disorder and the challenges surrounding the proposal of a new therapeutic is essential to developing disorder-specific therapeutic and management strategies based on the precision psychiatry concept.

In a clinical setting, depression is formally diagnosed using the term major depressive disorder (MDD). MDD is a neuropsychiatric disorder involving a plethora of heterogeneous phenotypes [78]. It belongs to the class of mood disorders and requires a qualified psychiatrist or clinical psychologist to diagnose an individual with clinical depression. The diagnosis of clinical depression requires the persistent presence of the cardinal symptoms of either depressed mood or anhedonia along with the other symptoms outlined in the diagnostic and statistical manual (DSM-5) for at least two weeks that significantly interfere with the functionality of the affected individual. Significant changes in weight or appetite, sleep disturbance, psychomotor changes, fatigue, a diminished ability to focus, and negative or suicidal thoughts are among other depressive symptoms listed in the DSM-5 [109]. These psychophysiological changes warrant clinical intervention when they are prolonged without reasonably precipitating external causes and severely affect an individual’s functioning [110]. A clinician’s collective judgment integrating a patient’s clinical history, physical examination, and laboratory findings is necessary to justify the formulated diagnosis and management strategies [38,43]. Antidepressants targeting the monoaminergic system are routinely prescribed as a first-line pharmacological treatment for depression. However, 20–30% of patients do not respond well to the existing pharmacotherapies, and the remission rate for monotherapy with the best antidepressants is merely 50.78% [111,112,113]. Most antidepressants were serendipitously discovered in the late 20th century by examining antidepressant properties exerted by drugs used to treat other non-psychiatric illnesses [114]. The primary therapeutic aim of antidepressants is to augment monoamine transmission. However, monoamine depletion has been shown to neither elicit depressive symptoms in healthy cohorts nor worsen the depressive symptoms in depressed patients [111]. Therefore, the efficacy and acceptance of these drugs have become a matter of debate due to their therapeutic latency, adverse side effects, and low remission rates, hence giving rise to the postulation of other possible biological underpinnings associated with the development of depression [110,115].

The exact etiopathophysiology of depression has yet to be established, partly owing to the highly heterogeneous nature of this mood disorder. Nevertheless, its neurobiological underpinnings have been understood to primarily involve altered neurochemicals, impaired stress response systems implicating neuroendocrine components, and neuroinflammation [78,116]. These neurobiological occurrences could be possibly ameliorated through the use of probiotics in depression [117]. Probiotics’ strain-specific and epigenetic potential counters the possible genetic elements involved in the development of depression. Probiotics targeting modifiable lifestyle and environmental factors, including stress and diet, provide room for a holistic approach toward clinical depression. Cumulatively, this microbial-based approach using probiotics complements the conceptual application of precision psychiatry in the clinical management of depression.

### 3.1. Neurobiological Bases

The understanding of the pathophysiologic basis of depression has broadened over the years, expanding to the neurobiological bases of depression beyond the monoamine hypothesis [110,118]. The long-standing monoamine theory has a limited scope to explain the occurrence of structural alterations in the brain regions and other biological findings reported in depressed patients [119]. Dysregulated hypothalamic-pituitary-adrenal (HPA) axis, neuroinflammation, and altered neurochemicals are some of the biological correlates of depression involving gut microbial-brain intertwining, which support the possible centric role of gut microbes in depression [2,110,120,121,122]. Chronic stress exposure leading to persistent elevation of stress hormones, mainly cortisol, has a culpable role in the dysregulation of the HPA axis. It has been elucidated that the MGBA is one of the pathways through which prolonged stress exerts such a consequential effect on the HPA axis, resulting in depressive symptoms [117,122,123]. One of the theoretical frameworks to explain this mechanism is by relating the internalized disruptive impact of stress on the gut microbial ecosystem, which augments an increased permeability of the intestinal barrier and subsequent activation of immune responses. The pro-inflammatory cytokines commonly associated with depression, tumor necrosis factor (TNF)-alpha, interleukin (IL)-1 and IL-6, and highly potent microbial antigens, lipopolysaccharides (LPS), possess the ability to cross the blood-brain barrier. This induces neuroinflammation and eventually enhances HPA axis activation.

Conversely, the hyperactivation of the HPA axis exerts harmful effects on the gut microbial ecosystem [117,124,125,126]. The stress-induced intestinal dysbiosis has also been linked to an altered intestinal fatty acid metabolism, which has consequential effects on adult hippocampal neurogenesis and neuroplasticity, exacerbating HPA axis hyperactivity [82]. These pathophysiologic events viciously affect one another, leading to the development of the depressive disorder. In terms of neurochemicals, altered levels of several neurotransmitters, including 5-hydroxytryptamine (5-HT), dopamine (DA), norepinephrine (NE), gamma-aminobutyric acid (GABA), and neurotrophin, brain-derived neurotrophic factor (BDNF), have been implicated in the occurrence of depressive disorders [111,127,128,129]. Gut microbes can modulate these neurotransmitter levels either directly via synthesis of neurotransmitters or indirectly via modulation of their precursors and stimulation of endocrine targets [14,129]. Neurotransmitters synthesized by the gut microbiota mediate gut-brain communication through signaling mechanisms involving afferent and efferent vagus nerve fibers [129,130]. BDNF, a neurotrophin, is critical to the brain’s neuroplasticity. BDNF maintains neuronal health and circuits involved in regulating emotions and cognition. In MDD patients and stressed animal models, neuroplasticity associated with altered BDNF levels has been reported to be profoundly disrupted [111,120]. Impaired BDNF neuroregulatory effect is also related to hippocampal atrophy, commonly reported in MDD patients [111,120,131]. The mechanism of antidepressant drugs has also been linked to enhanced regional expression of BDNF [132].

Most of the anti-depressive potential of probiotics was established in pre-clinical studies using mouse or rat models. In the pre-clinical setting, environmental stress and genetic manipulation are employed to mimic phenotypes akin to those of depressed humans [114,133]. The behavioral outcomes mimicking the antidepressant effects include improved mobility in the forced swim test, increased sucrose preference, and reduced latency in the novelty-induced hypophagia test [134,135,136]. The commonly reported biomarkers of depression include serum corticosterone levels (cortisol levels in humans) to assess the involvement of the HPA axis, pro-inflammatory cytokines (TNF-alpha, IL-1, and IL-6), brain-derived neurotrophic factor (BDNF), and neurotransmitters (5-HT, DA, NE, and GABA). Structural and functional assessments of the critical frontolimbic regions, amygdala, and hippocampus, are done using magnetic resonance imaging (MRI) [14,111,114,137,138,139].

*L. helveticus* R0052 and *B. longum* R0175 are frequently studied combined probiotics that are known to demonstrate significant anti-depressive potential in animal and human models [140,141,142,143,144,145,146]. This combination has been proven to attenuate the HPA axis and the autonomic nervous system’s (ANS) response to chronic stress. This was reflected by a decrease in plasma levels of the stress hormones’ corticosterone, adrenaline, and noradrenaline, in depression models of rodents [140,141]. This combination has also been shown to significantly restore colonic epithelial integrity and reduce gut permeability. It prevents degradation of tight junction proteins in the colonic mucosa, augments adult hippocampal neurogenesis, and restores synaptic plasticity to counter the stress-mediated insults involving the HPA axis [141,142,144]. In clinical studies, the same combination of probiotics markedly improved depressive scores and attenuated HPA axis hyperactivity, as evidenced by reduced cortisol levels [140]. The administration of *L. paracasei* CCFM1229 and *L. rhamnosus* CCFM1228 over six weeks in a chronic unpredictable stress model of mice yielded significant improvements in behavioral and neurobiological outcomes of depression. Both of these strains elevated 5-HT concentrations in the prefrontal cortex and BDNF levels in the hippocampus. The same study showed that L. paracasei CCFM1229 also reduced serum corticosterone levels, whereas *L. rhamnosus* CCFM1228 exerted no similar effect. These neurobiological outcomes were associated with reduced xanthine oxidase activity in the cerebral cortex and correlated to the beneficial modulatory effect of the probiotic strains on the host gut microbiome [139].

Most pharmacological treatments in MDD target the modulation of neurotransmitter activity in the brain [147]. *Lactobacillus* and *Bifidobacterium* strains are the most prominent probiotics associated with enhanced neurotransmitters, whose mechanisms are similar to antidepressant drugs [2,26,129]. In pre-clinical models of depression, *L. paracasei* PS23, *L. helveticus* NS8, *B. longum*, and *L. rhamnosus* were associated with increased hippocampal 5-HT levels [148,149,150]. *L. plantarum* PS128 has been shown to elevate 5-HT and DA levels in the striatum [151]. *B. infantis* was associated with decreased NA levels [152]. Regarding GABA, Bifidobacterium strains of human gut origin have been identified as the most significant contributors to this neurotransmitter, followed by *Lactobacillus* strains [153,154]. Among these strains, *L. plantarum* 90sk and *B. adolescentis* 150 have been ascertained as efficient GABA manufacturers with anti-depressant effects similar to fluoxetine [153]. Recent systematic reviews and meta-analyses have concluded that *Lactobacillus* and *Bifidobacterium* probiotics have the most significant effect on the augmentation of BDNF levels in patients with depression. *L. helveticus* R0052 and *B. longum* R0175 are the most commonly studied probiotics associated with such an effect on the BDNF levels in depression [155,156,157].

A clinical trial of the probiotic *B. longum* NCC3001 in individuals with irritable bowel syndrome utilized functional MRI to capture the brain activation pattern in the front-limbic regions, amygdala, and hippocampus. Administering the *B. longum* over six weeks lessened limbic reactivity to negative emotional stimuli while improving depressive scores [158]. Several other probiotic studies have presented the antidepressant potential of probiotics primarily through behavioral outcome measures [97,145,146,159]. *L. plantarum* 286, a probiotic of non-human origin, significantly improved depressive-like behavior in mouse models [98]. Combined probiotics (*L. helveticus* and *B. longum*; *L. acidophilus*, *L. casei*, and *B. longum*) administration over eight weeks in patients with MDD significantly improved depressive scores, evaluated using the Beck depression inventory (BDI), compared to placebo [146,159].

### 3.2. Genetic Bases

The advancement in genomic analysis has encouraged the reporting of probiotic designations with their strain type since 2010. The varying mechanisms of action on host pathogens, gut epithelial integrity, gut dysbiosis restoration, and immune response regulation are attributable to the distinct properties unique to each strain. Identifying probiotic strains allows the exploration of the disease-specific efficacy of probiotics, thus catering to the optimization of probiotic use [6,160]. In a pre-clinical study, the independent administration of *L. plantarum* 286 and *L. plantarum* 81 of non-human origin strains over 30 days yielded different outcomes, where the former exerted significant anti-depressive effects. However, no similar effect was seen in the *L. plantarum* 81 group, thus implying the strain-specific efficacy of probiotics [97]. On the other hand, administering *L. plantarum* 299v, a human-origin strain, over 8 weeks in patients with MDD significantly improved cognitive function. This was linked to decreased kynurenine levels in these patients, while no changes were observed in plasma levels of pro-inflammatory cytokines and cortisol [126]. Another study evaluated the behavioral and biochemical outcomes of different probiotic strains of *L. paracasei*, *L. rhamnosus*, *L. helveticus*, and *L. reuteri*. Only *L. paracasei* CCFM1229 and *L. rhamnosus* CCFM1228 exerted significant anti-depressive effects with minimally different biochemical outcomes. These strains produced different neuroregulatory impacts on genes implicated in neuroplasticity and the gut microbiota-inflammasome pathway. No optimal anti-depressive effects were elicited by various strains of *L. helveticus* and *L. reuteri* [139,161]. The strain-specific probiotic mechanisms provide an understanding of the possible genetic bases of microbial agents in the amelioration of depression. This occurs by regulating central gene expression in the key brain regions responsible for mood regulation and cognitive functions, as well as modulation of inflammatory and immune gene expression. These regions include the hippocampus, amygdala, prefrontal cortex, and hypothalamus. This ultimately contributes to improved neurogenesis and neuroplasticity within these brain regions that otherwise have a pathophysiologic association with depressive disorder [27,82,141,162,163,164].

Adult neurogenesis in the dentate gyrus of the hippocampus has been strongly correlated with a causal factor of MDD and plays a role in the attenuation of the HPA axis. Hippocampal atrophy associated with reduced adult neurogenesis has been commonly reported in MDD patients [82,165,166]. One of the causative mechanisms linked to reduced hippocampal neurogenesis is an impairment in the endocannabinoid (eCB) signaling system involving the cannabinoid receptor (CB1), which is instrumental in the regulation of hippocampal adult neurogenesis [167,168]. A genetic study in MDD patients has also shown an increased frequency of a mutant allele for the CB1 receptor gene, CNR1 [169]. In essence, the host gut microbial dysbiosis affects the intestinal fatty acid metabolism, leading to a paucity of precursors, namely arachidonic acid (AA), thus downregulating hippocampal levels of 2-arachidonoylglycerol (2-AG), required in the activation of CB1 [82,170]. *L. plantarum*^WJL^ administration in a mouse model of depression associated with dysbiotic intestinal microbiota significantly ameliorates depressive-like behavior by normalizing reduced adult hippocampal neurogenesis. This was linked to the microbial restoration of fatty acid metabolism, which augmented hippocampal eCB precursors, 2-AG levels, and subsequent upregulation of CB1 receptors [82]. *L. helveticus* R0052 and *B. longum* R0175 combination probiotics augmented adult hippocampal neurogenesis, leading to improved synaptic plasticity within the hippocampus and hypothalamic regions in animal models of depression. This genetic basis is correlated with the increased expression of several hypothalamic genes involved in neurotransmission and synaptic plasticity, thus enhancing the neuronal network within the hypothalamus to exert anti-depressive effects [141].

#### Epigenetics

Depression has been described as a pleomorphic illness that arises from gene-environment interactions, with genetic heritability only accounting for 40% [171,172]. The epigenetic mechanism explains how environmental factors such as stress and diet influence the neurobehavioral outcome of depression and the means of exogenous intervention such as probiotics [173,174,175]. However, no studies have exclusively explored and demonstrated the epigenetic potential of probiotics in depression, considering the recentness of the epigenetic branch of genetic studies. Nevertheless, the existing studies provide some solid footing for understanding the possible epigenetic basis of probiotics in depression [2,164,173,176,177,178]. Epigenetic mechanisms introduce long-lasting, heritable, yet reversible phenotypic changes without involving genotypic alteration through methylation of deoxyribonucleic acid (DNA), histone modification, and non-coding ribonucleic acid (RNA) [2,175,176,177,178,179,180,181]. DNA methylation is predominantly associated with a suppressive effect on gene transcription, whereas histone acetylation is associated with a gene induction effect [182]. Environmental factors, including gut microbes and related metabolites, alter the host’s epigenetic signatures, inducing varying responses and favorable or deranged outcomes involving an inflammatory cascade due to the alteration within the gut-brain axis. Gut-modulating agents such as probiotics act as epi-drivers to exert beneficial modulatory epigenetic effects on the host epigenome [175,181,183,184]. Probiotics exert prophylactic and nullifying epigenetic effects to counter the adverse alteration of the host’s epigenetic signatures [164,184]. A recent study demonstrated the strain-specific epigenetic potential of lactobacilli probiotics, *Limosilactobacillus fermentum* MTCC 5898 and *Lacticaseibacillus rhamnosus* MTCC 5897, on DNA and histone modifiers independently and following a challenge test using an inflammation-stimulating, opportunistic pathogenic commensal, *Escherichia coli* ATCC 14849 [164]. *E. coli* has also been shown to disrupt intestinal epithelial integrity [185]. The mRNA gene expression of DNA and histone modifiers in the host intestinal epithelial cells was assessed to determine the epigenetic ability of the lactobacilli probiotic strains using an in vitro model of intestinal inflammation and permeability, Caco-2 cells. *Limosilactobacillus fermentum* significantly increased the mRNA expression of both epigenetic modifiers in the Caco-2 cells independently at 12-h of incubation, while *E. coli* reduced the mRNA expression. The mRNA expression was even higher following a 12-h challenge test with *E. coli* after a 12-h pre-treatment with *Limosilactobacillus fermentum*, implying the epigenetic signatures of this probiotic strain. However, no similar effects were exerted by *Lacticaseibacillus rhamnosus* [164]. In another study, the same lactobacilli probiotic strains were shown to significantly reduce histone 3 and histone 4 acetylation at 6-h incubation independently and in combination when *E. coli* were excluded, competed with, or displaced by these probiotic strains. On the other hand, DNA-methylation was significantly enhanced following the *E. coli* challenge test following pre-treatment of Caco-2 cells with lactobacilli strains in the exclusion assay and competition assay without pre-incubation by these probiotics. The predetermined dose of the strains used for a 6-h incubation period was based on the ability of these indigenous microbes to optimize the expression of tight junction genes associated with intestinal epithelial barrier functions [184]. These studies substantiate the power of probiotic bacteria to modulate host epigenetic patterns through DNA-methylation and histone modifications to mediate either prophylactic or nullifying effects to counter the deranging epigenetic exertion by pathogenic microbes associated with inflammation and barrier functions of the gut epithelial cells.

Other studies have demonstrated the epigenetic potential of probiotics through the regulation of BDNF and histone deacetylase (HDAC) inhibition by its metabolites consisting of short-chain fatty acids (SCFAs), mainly butyrate, propionate, and acetate [2,175,181]. HDAC inhibition acts at the level of the hippocampus and amygdala and has an activation role on histone acetylation, which is associated with the consolidation of learning and memory [186]. Butyrate is a potent HDAC inhibitor, followed by propionate and acetate, whose mechanisms have been compared to antidepressants [187,188,189]. These SCFAs can penetrate the blood-brain barriers and exert modulatory effects on hippocampal gene expression, neurochemicals (BDNF levels, GABA, and 5-HT production), and neuroinflammation through HDAC inhibition [129,181,190]. This is consistent with the commonly reported reduced abundance of butyrate-producing gut bacteria of the *Faecalibacterium* and *Coprococcus* genera in patients with MDD [191,192]. Multi-strain probiotic supplementation for 28 days, consisting of *B. bifidum*, *B. lactis*, *L. acidophilus*, *L. casei*, *L. paracasei*, *L. plantarum*, *L. salivarius*, and *L. lactis*, significantly augmented butyrate-producing gut bacteria *Coproccocus* 3 and *Ruminoccocus grauvanii* and improved depressive outcome in depressed patients compared to the placebo arm [193].

### 3.3. Environment and Lifestyle Bases

#### 3.3.1. Stress

Stress is often accompanied by environmental triggers and life experiences, thus implying that stress is a modifiable lifestyle component of precision psychiatry. Stress has adverse effects on various physiological processes in the human body. Prolonged stress is a significant risk factor for the development of depression [173,194]. Chronic stress imposes vicious consequences on the neuronal structure and functionality through its disruptive effects on the integrity of the intestinal barrier, escalation of inflammatory responses, and a maladaptive stress-response system involving the HPA axis. It reduces hippocampal and hypothalamic expression of glucocorticoid receptors and suppresses hippocampal neurogenesis, which ultimately contribute to the pathogenic occurrence of depressive disorder [111,117,141,194]. Persistently elevated levels of glucocorticoids, particularly cortisol, impose detrimental alterations to the critical frontolimbic structures and neurocircuits, which are instrumental in regulating emotions and rewarding behaviors [195]. Therefore, stress is a vital lifestyle element to be considered in the prevention and management strategies of MDD. The environmental triggers influence the extent of internalization of stress-mediated responses, thus warranting necessary, timely intervention, particularly in individuals with biological and genetic susceptibility to the development of MDD [196].

Pre-clinical studies of probiotics routinely use rodent stress models to demonstrate probiotics’ anti-depressive outcomes [197]. However, in the human context, stress is a risk factor associated with MDD. Therefore, the efficacy of probiotics is evaluated based on psychological outcomes in healthy cohorts or stressed individuals to attest to their preventive role in MDD. In 66 healthy human volunteers, probiotic supplementation consisting of *L. helveticus* R0052 and *B. longum* R075 over 30 days significantly improved their mood and overall psychological well-being assessed through a few self-reported measures, including the hospital anxiety and depression scale (HADS). It reduced free urinary cortisol levels, indicating possible attenuation of the HPA axis [140]. In students aged between 18 and 24 years old facing examination stress, a 28-day intervention with a multi-strain probiotic consisting of *Bacillus coagulans* Unique IS2, *L. rhamnosus* UBLR58, *B. lactis* UBBLa70, *L. plantarum* UBLP40, *B. breve* UBBr01, and *B. infantis* UBBI01 not only improved the stress outcome but also significantly reduced serum cortisol levels [198]. In another study, a multi-strain probiotic consisting of *B. bifidum* W23, *B. lactis* W52, *L. acidophilus* W37, *L. brevis* W63, *L. casei* W56, *L. salivarius* W24, *L. lactis* W19, and *L. lactis* W58 significantly improved cognitive reactivity to either acute stress or sad mood in healthy individuals [199,200]. Administration of *L. plantarum* P-8 over 12 weeks in stressed adults alleviated stress, modulated gut microbiota, increased abundance of *B. adolescentis*, *B. longum*, and *F. prausnitzii* and enhanced levels of microbial neuroactive metabolites SCFA, AA, and GABA [201]. These studies demonstrate distinct stress-alleviating effects of probiotics, thus indicating their usefulness as a preventive intervention in MDD.

#### 3.3.2. Diet

The human gut is exposed to various pathogenic and commensal microorganisms as a double-edged sword. As long as the intestinal immune system can discriminate between these microbes’ beneficial and pathogenic activities and elicit the appropriate response, the human host will continue to reap the positive physiological outcomes of this cross-talk between the gut microbes and the immune system. However, if the gut microbial balance is disturbed, homeostatic regulation will be severely affected, leading to the manifestation of immune responses [202,203]. Diet is an environmental and lifestyle factor imperative to maintaining intestinal homeostasis and immune regulation. The current concept of psychobiotics incorporates a microbiota-targeting diet as a psychobiotic agent that modulates gut-brain communication to exert beneficial mental health outcomes [174,204]. In this context, probiotics are “traditional” psychobiotic agents that exert pronounced anti-depressive effects similarly. Fruits and vegetables rich in prebiotic fiber, fermented foods, whole grains, and legumes are constituents of a psychobiotic diet that have been proven to ameliorate depressive symptoms [174]. SCFAs, which are microbial metabolites produced through the fermentation of dietary fibers, have critical mediating and epigenetic roles within the MGBA in the modulation of neurotrophic factors and neurotransmitters, neuronal transcription, inflammation, and gut barrier functions [175,180,181]. One of the recent pioneering studies that evaluated the impact of a psychobiotic diet on perceived stress and microbiota composition in healthy adults demonstrated a significant reduction in perceived stress with minimal changes to the microbial composition. It was further inferred that the stress-alleviating effect of the intervention is dose-dependent [174]. Several other studies in healthy adults investigated the impact of dietary interventions and high-fibre, fermented foods on microbial diversity and the immune system and demonstrated profound changes in microbial diversity, increased abundance of Lactobacillus and Bifidobacterium, and reduced inflammatory markers [205,206,207]. These studies substantiate the influence of diet on gut microbial diversity and mental health, thus making it an ideal target for preventing MDD.

## 4. Potential Microbial-Based Approach in the Clinical Management of Depression

Although conventionally, the diagnosis of MDD lacks diagnostic tests in terms of clinical biomarkers, the microbiome field unfolds a new dimension in this context by incorporating the possible microbial-based biomarkers and accompanying neuroimaging assessments. This caters to a personalized treatment approach using probiotics as either adjunct or stand-alone treatment modalities [208]. The biomarkers commonly associated with the pathomechanism of depression related to microbial etiopathogenesis include pro-inflammatory cytokines, cortisol, BDNF, and kynurenine. These can be easily evaluated through the analysis of a patient’s blood sample [138,209]. BDNF evaluation also correlates with suicide risk, while kynurenine levels correlate with cognitive function [111,126]. The advancement in our understanding of the complex, bi-directional interaction between the human brain and gut microbiota since the early 2000s may herald a new era in more clearly defined diagnostic criteria, removing some of the ambiguity in identifying MDD [14].

Magnetic resonance imaging is one of the most commonly advocated neuroimaging tools to capture structural and functional changes within the different regions of the human brain. These findings are correlated with gut microbiota profiling and neuropsychological scorings [53,158,210]. Several studies have substantiated the neuroimaging findings in MDD patients, which commonly involve structural and functional assessments of the vital frontolimbic regions and circuits [137,211,212]. Hippocampal atrophy is a widely reported hallmark in patients with MDD [195,213]. Hippocampal atrophy is associated with reduced adult hippocampal neurogenesis due to impaired HPA axis linked with microbial dysbiosis in depressed cohorts [82]. The frontolimbic assessment has also been suggested as a valuable predictor of suicide risk in individuals with depression [214]. Therefore, functional and structural assessments of the key limbic regions (hippocampus and amygdala) and neuronal circuitry in these regions using MRI are potential diagnostic neuro-biomarkers. This may cater to a personalized probiotic choice that complements conventional clinical intervention in MDD [211,215].

Machine learning provides a comprehensive, integrated, futuristic management approach in clinical depression [216], unfolding a new horizon in the healthcare sector by incorporating artificial intelligence to develop computational software that allows objective measurement of integrated large-scale datasets to complement clinical management of health conditions. Machine learning provides an integrated approach to patient care by tackling various clinical aspects, including diagnosis prediction, treatment selection, patient compliance, and administrative tasks. Ultimately, this tailors to a personalized patient care and precision medicine approach [104,217]. Converging this application in the presented context of probiotics in clinical depression, machine learning paves the way for integrating microbial bases in depressive disorder from the consortia of the characteristic, endophenotypic, neurobiological, and genetic censuses to optimize management and treatment options using probiotics. Although this remains a futuristic vision, a handful of studies have begun to explore the machine learning application in microbiome, probiotics, and precision psychiatry [104,106,218,219,220,221].

**Figure 1:**
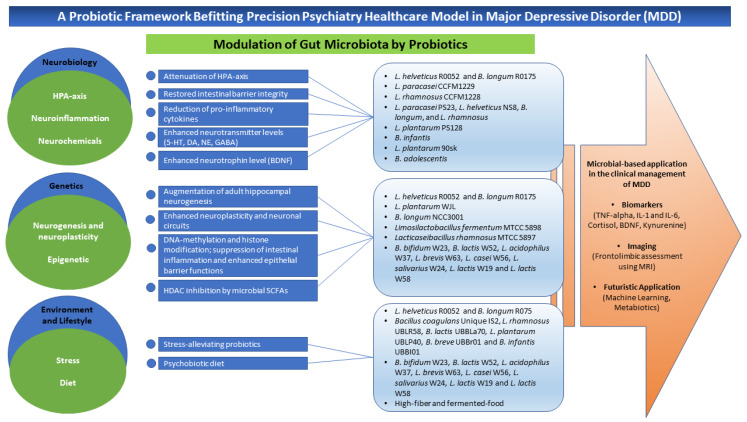
A probiotic framework befitting the precision psychiatry healthcare model in MDD. This framework represents three main components of precision psychiatry: neurobiological bases, genetic bases, and environment and lifestyle elements of MDD that involve microbial intertwining. Modulation of gut microbiota is the rudimentary anti-depressive mechanism of probiotics. Some commonly explored probiotics of *Lactobacillus* spp. and *Bifidobacterium* spp., either as a single strain or multi-strain, exert anti-depressive mechanisms by acting on different pathophysiologic mechanisms implicated in depressive disorder. Stress and diet are mainly modifiable targets in the prevention of MDD. The microbial-based applications in the clinical management of MDD include some potential biomarkers obtainable through a patient’s blood analysis, assessment of vital frontolimbic regions in the brain using MRI, and integration of machine learning and development of depression-specific metabiotic as part of futuristic vision within this context.

Metabiotics, identified as the evolutionary concept building on the foundation of knowledge of probiotics, is another microbial-based application in the context of clinical depression. It involves engineering the human gut microbiota through natural selection buffered using probiotics or synthetic manufacturing to produce targeted health outcomes. Metabiotics refers to metabolites, structural components, and/or signaling molecules of probiotic bacteria with an identifiable chemical structure that can optimize host physiological functions and exert regulatory effects on metabolic and/or behavioral outcomes associated with the activity of the host microbiota. This concept caters to the selection of novel therapeutics using probiotics with known chemical formulas, dosage, safety profile, and durability, thus expanding the microbial-based applications not only in the psychiatric domain but across a vast domain of human health [27,222]. The probiotic framework befitting the psychiatric healthcare model in MDD has been summarized in Figure 1 below.

## 5. Limitations and Translational Gaps

In medical research, the transferability of data from animal models to humans is often the most crucial element to consider in terms of developing and establishing a proposed clinical intervention. The promise of probiotics in treating depression has been demonstrated mainly in pre-clinical settings using either rat or mouse models. The gut microbiota of rats and mice resembles the human gut microbiota, thus making them ideal models for microbial manipulation using probiotics. However, inducing depressive phenotypes in the pre-clinical models is a one-dimensional exploration of depression compared to a human cohort, considering the highly heterogeneous nature of the depressive disorder and human gut microbiota, coupled with the varying host and environmental variables in a human setting. The inconsistent response toward probiotics in pre-clinical models suggests a need for standardization in gut microbiota composition and conditioning in research models to help understand the influence of the gut microbiota on clinical manifestations. However, it is impractical to standardize the human gut microbiome [40]. Despite these limitations, clinical trials of probiotics reporting promising results in depression models have been emerging since the early 2000s, attesting to the potential efficacy of probiotics in alleviating depression [8]. Significant gut dysbiosis has been established in depressed human subjects compared to healthy subjects, which has paved the way to utilize probiotics as humans’ gut microbiota-modulating agents to exert the desired anti-depressive effects. Although scientists have yet to define a conclusive pattern of gut dysbiosis in depression, the ability of probiotics to exert anti-depressive effects primarily through gut microbiota modulation reflects the dynamicity of probiotics’ mechanism of action notwithstanding the hosts’ distinct indigenous microbial compositions [40,223,224,225]. The observable discrepancies and inconsistencies among the findings of various studies on the gut microbial profile of depressed subjects, mainly in terms of identified bacterial genus and species, may be due to the varying age groups, health status, lifestyle, and receptivity of the study subjects as well as the technical aspects of the study [88,226]. The variability in terms of gut microbial profiles may account for the subjects varying degrees of responsiveness to probiotics—again providing a tantalizing suggestion that further characterization of specific microbiome profiles may lead towards customizable, personalized probiotic-based therapy for MDD.

Additionally, before probiotics can be certified and validated as a clinical treatment modality, many aspects must be considered, including factors associated with probiotics (type of strains, dose, administration method, clinical outcome), host factors, and practical applicability and feasibility in a clinical setting [227]. Selecting probiotics specific to depression is challenging considering the scarcity of available studies, particularly clinical trials. The beneficial properties of a probiotic bacteria may differ between strains of the same bacterial species and target health conditions. The categorical factors to consider in determining the appropriate probiotics specific to depression include the probiotic strains, the nature of the depressive disorder, and host factors [6]. The type and combination of strains and the origin of the strains (human versus non-human) account for the optimum efficacy of probiotics. The human-origin strains confer better adaptability to potentially hostile physiological environments due to the varying degrees of pH down the digestive tract compared to the non-human-origin strains [47,228]. The most recent studies have further concluded that combined strains have better health outcomes than single strains of probiotics. Cumulatively, combined probiotic strains of *Lactobacillus* and *Bifidobacterium* spp. remain an ideal option for their significant anti-depressive potential [20,26]. Although no clinical trials of probiotics in patients with MDD have thus far reported any adverse events associated with using probiotics, it is worth evaluating the risk of introducing probiotics in individuals who are immunocompromised and critically ill. The potential threat of probiotics to cancer patients, pregnant women, infants, and elderly cohorts is equally worthy of attention. Some of the reported adverse effects of probiotics include gastrointestinal disturbances, skin complications, and septicaemia in a worst-case scenario [229], suggesting the need for caution and further evaluation before introducing them to a vulnerable patient population.

In terms of MDD, evaluation by medical personnel of the overall clinical manifestations and potential biomarkers within the arrays of the microbiome is necessary to determine the appropriate probiotics to tackle this highly heterogeneous disorder. The most challenging factor to consider when selecting probiotics would be the host factors, considering the dynamic microbial changes that occur across an individual’s lifespan due to aging, lifestyle, geographical influences, host immune status, and existing comorbidities. This may influence the degree of responsiveness to probiotics in an individual [2]. Additionally, the lack of revenue and resources may be possible barriers to implementing the proposed microbial-based assessments, particularly in remote clinical settings.

## 6. Conclusions

In theory, and based on the limited literature currently available in this relatively new field, probiotics appear to have tremendous potential in the treatment of MDD. However, seeking to validate probiotics usage for this indication will be challenging and elusive, considering the inherently ambiguous nature of this enigmatic branch of medicine and the scarcity of probiotic studies in clinical depression. Nevertheless, the increased acceptance of precision psychiatry promises a paradigm shift in approaching psychiatric disorders. The current research directive in the field of probiotics centers around identifying probiotics’ strain-specific and disease-specific efficacy, which in turn promotes the personalization of probiotics. At the present stage, in the context of clinical depression, it is worth considering probiotics as an adjunct treatment in general and a stand-alone treatment in patients with mild MDD who may not necessarily require conventional pharmacological treatment. It may also be a preventive intervention for individuals at risk of developing MDD. It has been proven that different strains of probiotics exert anti-depressive potential via distinct mechanisms. Hence, it seems only fitting that scientists and industrialists consider developing probiotic strains that effectively ameliorate depression by tackling different neurobiological and genetic bases of this disorder. The presented probiotic strains could be utilized as starter strains for industrial development and manufacturing of metabiotics to address the gap in determining the selection of target novel probiotics. Acknowledging that the existing studies are only the tip of the iceberg and considering the vast possibility of probiotics in depression, more studies are imperative, especially in the patient cohort, to promote and expand the use of probiotics in clinical settings in parallel with the precision psychiatry approach. More implementation research is also required to bridge the existing translational gap and encourage practical application in real-world medicine. Based on all available evidence, the authors embrace the microbial-based, revolutionary approach in patient care and emphasize the collective role of clinicians, scientists, and industry in mediating this consolidated effort to highlight the therapeutic potential of probiotics in clinical depression.

## Data Availability

Not applicable.
